# Identification of Activity Breaks Using Accelerometry

**DOI:** 10.3390/s26196194

**Published:** 2026-09-30

**Authors:** Meredith Peddie, Jennifer Gale, Hannah Tamblyn, Jillan Haszard

**Affiliations:** 1 Department of Human Nutrition, University of Otago, 362 Leith Street, North Dunedin, Dunedin 9016, New Zealand; hannahschat8@gmail.com; 2 Faculty of Health and Environmental Sciences, Auckland University of Technology, 55 Wellesley Street East, Auckland Central, Auckland 1010, New Zealand; jen.gale@aut.ac.nz; 3 Haszard Biostatistics, Department of Nutrition, University of Auckland, 24 Princes Street, Auckland 1010, New Zealand; jill@haszardbiostatistics.co.nz

**Keywords:** sedentary behaviour, physical activity, actigraphy

## Abstract

**Highlights:**

**What are the main findings?**

- Using Lasso regression and multi-site accelerometry (wrist, hip, thigh), the study successfully developed an algorithm to objectively identify short, frequent activity breaks in free-living conditions. Algorithms were also developed for each accelerometer individually.
- The algorithm showed agreement with self-reported data, with no mean difference in the number of activity breaks detected and only a small overestimation of break duration (~0.9 min).

**What are the implications of the main findings?**

- External validation confirmed robust performance, supporting the algorithm’s utility for quantifying activity breaks in free-living studies and informing interventions targeting sedentary behaviour.

**Abstract:**

Laboratory studies show that performing ~2 min of activity every 20–30 min, known as activity breaks, is associated with improved cardiometabolic health outcomes. However, objectively identifying these activity breaks in a free-living setting remains challenging. This study aimed to develop and validate an algorithm to detect activity breaks using accelerometer data. Thirty-one healthy adults (mean (SD) age 33 (12) y, 71% female) wore three accelerometers (ActiGraphs on the wrist and hip; ActivPAL on the thigh) while completing eight activity breaks, approximately 30 min apart. Participants self-recorded activity break start and stop times. Lasso regression with an ‘activity break’ definition was used to develop an algorithm that used all three accelerometers and for each accelerometer individually, using two-thirds of participant data for development and the remaining third for testing. External validation was undertaken using an independent dataset. For the three-device algorithm, the mean difference between predicted and reported activity breaks was 0.0 (95% CI: −1.7, 1.7), while predicted break duration was, on average, 0.9 min longer than the reported duration (95% CI: 0.2, 1.6). These findings suggest the algorithm accurately estimates the frequency and duration of activity breaks and may be useful for quantifying this behaviour in free-living studies.

## 1. Introduction

Sedentary behaviour, particularly when it is accumulated in prolonged, uninterrupted bouts, is associated with a poorer cardiometabolic profile [1,2], a higher incidence of disease [3], and mortality [4]. Experimental studies in adults have demonstrated that interrupting prolonged sitting by performing short (2–3 min), regular (every 20–30 min) bouts of activity (henceforth referred to as regular activity breaks) reduces postprandial glucose and insulin concentrations [5] and may also reduce postprandial triglyceride concentrations [6] and blood pressure [7], increase blood flow [8], and extend subsequent sleep duration [9]. Additionally, performing regular activity breaks may be a more accessible way of introducing physical activity into the day of those who are most at risk of poor health [10].

Interruptions in sedentary time (defined as a transition from standing to sitting) can be successfully identified with an ActivPAL accelerometer placed on the thigh [11]; however, ActivPALs tend to perform less well when identifying activity by intensity [12]. Additionally, ActiGraph or actiheart devices have been found to overestimate the number of breaks in sedentary time (commonly defined as any time counts per minute exceed 100, or 150 for longer than a minute) [13,14]. Therefore, it is not that surprising that objectively identifying intentionally performed activity breaks that match the intensity and frequency of the laboratory-based study remains challenging. In our recent study [15] we found a large disparity between changes in self-reported activity breaks and changes in activity breaks identified in accelerometer data (when an activity break was defined as an increase in counts per minute to greater than 1000 cpm, sustained for at least a minute, when preceded by at least 15 min of sedentary time [15,16]). Accurate objective measurement of activity breaks is critical to allow assessment of adherence to intervention protocols.

Therefore, the objective of this study was to develop and validate an algorithm to identify activity breaks using data from accelerometers placed on the hip, wrist, and thigh from a sample of participants who undertook intentional activity breaks approximately every 30 min. Internal (as part of the development) and external validation (in a separate sample of participants involved in an intervention study) were also undertaken.

## 2. Materials and Methods

### 2.1. Participants

Adults (*n* = 31) aged 18 years and over were recruited for the algorithm development study via convenience sampling. Eligible participants were required to be able to ambulate unaided (i.e., not using a wheelchair, crutches, or other assistive devices), be free from conditions or disorders that impact movement patterns (e.g., Parkinson’s, Essential Tremor, Cerebral Palsy), and have answers to the Physical Activity Readiness Questionnaire (PAR-Q) indicating that participating in physical activity was medically appropriate. All participants provided electronic informed consent, and the study was approved by the University of Otago Ethics Committee (Health; H23/104).

### 2.2. Protocol

Before being fitted with the accelerometers, participants completed a brief online demographic questionnaire (REDCap software, Research Electronic Data Capture, production server version 13.10.0, Vanderbilt University, Nashville, TN, USA) and had their height and weight measured following standard procedures for descriptive purposes. Each participant was fitted with three accelerometers. Two ActiGraph GT3x+ (Ametris, formerly ActiGraph LLC, Pensacola, FL, USA) accelerometers (initialised to record at 30 Hz), worn on elasticated straps over the non-dominant wrist (determined via self-report) and right hip, and one ActivPAL (PAL Technologies Limited, Glasgow, Scotland) accelerometer, which was adhered to the front of their right thigh with a waterproof hypoallergenic dressing. When fitting the accelerometers, the research assistant checked that the time on the computer used to initialise the accelerometers matched the time on the device the participants used to mark time (e.g., watch or mobile device). Any deviations were noted to ensure the times of activities recorded by the participant would match the time-stamped data recorded by the accelerometer.

Once the accelerometers were fitted, participants were instructed to return to their usual daily routine. However, during this time they were asked to remain mostly sedentary, but to perform eight bouts of 2–3 min of activity approximately every 30 min (a total duration of ~4.5 h). Immediately upon completion of each activity break, participants were asked to record, on a paper recording sheet, the exact start and finish time, a brief description of the type of activity and an indication of whether they considered the intensity of the activity to be light, moderate or vigorous. A list of suggested activity types was provided to the participants, including body weight resistance exercises, walking on the spot or around the house, jumping jacks, aerobics, or dancing. Participants were encouraged to select individual or a combination of activities from the list to perform in each activity break, or to come up with their own activities, but were discouraged from using stretching or static activities (e.g., planks or wall sits). Once the participants completed their eighth and final activity break, they were encouraged to continue wearing them for at least a further 30 min, at which point they could remove them and return them to the research team the following day.

### 2.3. Data Processing

Data from participants were only included if they had complete data (that included recording 8 activity breaks) from at least one accelerometer (thus there was no need to define non-wear criteria or valid days of data). All participants had complete data from both ActiGraphs, but three ActivPALs malfunctioned, meaning no data was collected from this device for three participants. Time-stamped activity count data from both the ActiGraph and ActivPAL were downloaded in 15 s epochs using ActiLife (ActiLife version 6.13.4) and ActivPAL (PAL: Physical Activity Logging Research Edition, version 7.2.38) software and imported into Stata 19.0 (StataCorp, College Station, TX, USA). All three files were then merged to create one file for each participant, and activity breaks were identified from recorded participant information and used to code each epoch as ‘1’ for the epochs when the activity breaks were known to happen (with all other epochs coded as ‘0’ for that variable, even if count data indicated movement).

### 2.4. Prediction Modelling

The sample was randomly split into a development sample (67%) and a test sample (33%). An observation was defined as a 15 s epoch. The activity break variable (coded from participant-recorded information) was used as the ‘reference’ against which the objective measures from the accelerometers would be compared. Nine predictor variables were included in the prediction modelling: The counts from each of the three axes from the three accelerometers. Summary outputs (such as vector magnitude) were not included.

Using the ‘pmsamplesize’ in Stata [17], and a conservatively low Cox-Snell R^2^ of 0.02, with nine parameters, we would need a sample size of at least 4000 epochs of data. Collecting data from at least 30 participants means we anticipated having over 30,000 epochs of data, which allows for a very large design effect of 7.5 to account for any influence of within-person clustering, which is expected to be low.

Lasso logit regression [18] with 10-fold cross-validation was used to generate a prediction equation for identifying epochs in activity breaks using the development sample, with participant ID included as a cluster variable. For replicability, the seed was set to 4724 (the date when modelling began). Lasso regression fits a range of models using different coefficients for the prediction variables and tests the models against the ‘gold standard’ using 10-fold cross-validation. The model (identified by a ‘shrinkage parameter’ called lambda) with the lowest mean deviance was selected. A cross-validation (CV) plot of the mean deviance for decreasing values of lambda was generated, and values of the CV mean deviance, the CV deviance ratio (an out-of-sample measure of goodness of fit, similar to R^2^), and BIC for knots in the modelling were generated (knots are values of lambda where the prediction variables in the model change).

### 2.5. Classification of Epochs

The selected model was used to predict values for each epoch. These values represented the probabilities from a logit model that the epoch was activity rather than an activity break. The cut point at which these probabilities identified activity from an activity break was determined using the Liu method [19], which balances sensitivity and specificity. The area under the curve (AUC) under the ROC curve was calculated and plotted. This cut point was then used to classify epochs as either ‘1’ (from an activity break) or ‘0’ (not from an activity break). It was expected that there would be several epochs classified as from an activity break that were not from a recorded activity break, as participants did move around outside of their recorded activity breaks.

To tidy up ‘rogue’ epochs (single 1s or 0s in a series), single 0s surrounded by a 1 before and after were converted to 1s, and then single 1s surrounded by a 0 before and after were converted to 0s. The order of these tasks was determined by assessing the AUC from the ROC curve, and the order reported here is the order that resulted in the highest AUC.

### 2.6. Classification of Activity Breaks

To align with the pattern of activity found to be associated with health benefits in laboratory-based studies, the *start* of an ‘active break’ was defined as the end of three consecutive 0s followed by three consecutive 1s and vice versa for the *end* of an active break. All epochs between the ‘start’ and ‘end’ were classified as 1 (active epochs).

Data were then collapsed using the ‘tsspell’ command in Stata [20], which reduced ‘spells’ of epochs into ‘active breaks’ or ‘sedentary bouts’, with a count of epochs and a sequence number for each. From this, as decided a priori based on previous experimental and/or observational work, all active breaks that were longer than 10 min (40 epochs) were reclassified as ‘physical activity’, because this is too long to be an ‘activity break’. Active breaks less than 1.5 min (6 epochs) were reassigned as sedentary, as these were too short to be an activity break, based on the duration of activity breaks tested in laboratory studies. Sedentary bouts were then reconsolidated, and bouts less than 5 min (20 epochs) were reassigned as ‘mixed’, because an activity break should break a period of uninterrupted non-active sedentary time. An ‘activity break’ was then defined if it was classified as an active break and was surrounded by sedentary bouts before and after.

The number of activity breaks per participant, and the mean length of these activity breaks were calculated and compared with the self-reported activity breaks using descriptive statistics and paired t-tests.

### 2.7. Test Sample

The proposed prediction equation and processing steps for defining activity breaks were then applied to the test sample, and comparisons with self-reported activity breaks were made.

### 2.8. Different Inputs

The methods described were then repeated using data from each accelerometer separately, to provide algorithms for each accelerometer. The total number of activity breaks using the different data inputs from both the development and test samples was plotted as box plots to compare the distributions against the self-reported activity breaks.

### 2.9. External Validation

Data collected in a separate study that was piloting an intervention where participants were being asked to perform activity breaks in a free-living setting was used to test the validity of the algorithm with paired t-tests and 95% limits of agreement. In this study (which was approved by the University of Otago Ethics Committee (Health; H23/105)), 20 participants (*n* = 18 female, mean (SD) age: 34 (23) y, BMI 33 (10) kg/m^2^) self-reported activity breaks and wore three accelerometers in a manner similar to that described here for two seven-day periods.

## 3. Results

A total of 31 participants involved in the algorithm development study provided accelerometry data, the majority of whom were female, with a mean BMI of 27 kg/m^2^ (Table 1). Over half (54%) of activity breaks were performed by walking, running or jogging, with nearly a third (30%) being resistance exercises and the remainder being made up of plyometric exercises (e.g., jumping jacks; 8%), using cardio equipment (e.g., a rowing machine; 6%), dancing or yoga (2%). Activity breaks were mostly performed at a self-reported light intensity (44%) and moderate (50%) intensity.

Data from the 31 participants were randomly split into two samples: the development sample (*n* = 21) and the test sample (*n* = 10). The number of epochs from the hip and wrist accelerometers was 48,363, with 35,553 in the development sample and 12,810 in the test sample. Three participants were missing data from thigh accelerometers (two in the development sample and one in the test sample).

### 3.1. Prediction Modelling

**Figure 1:**
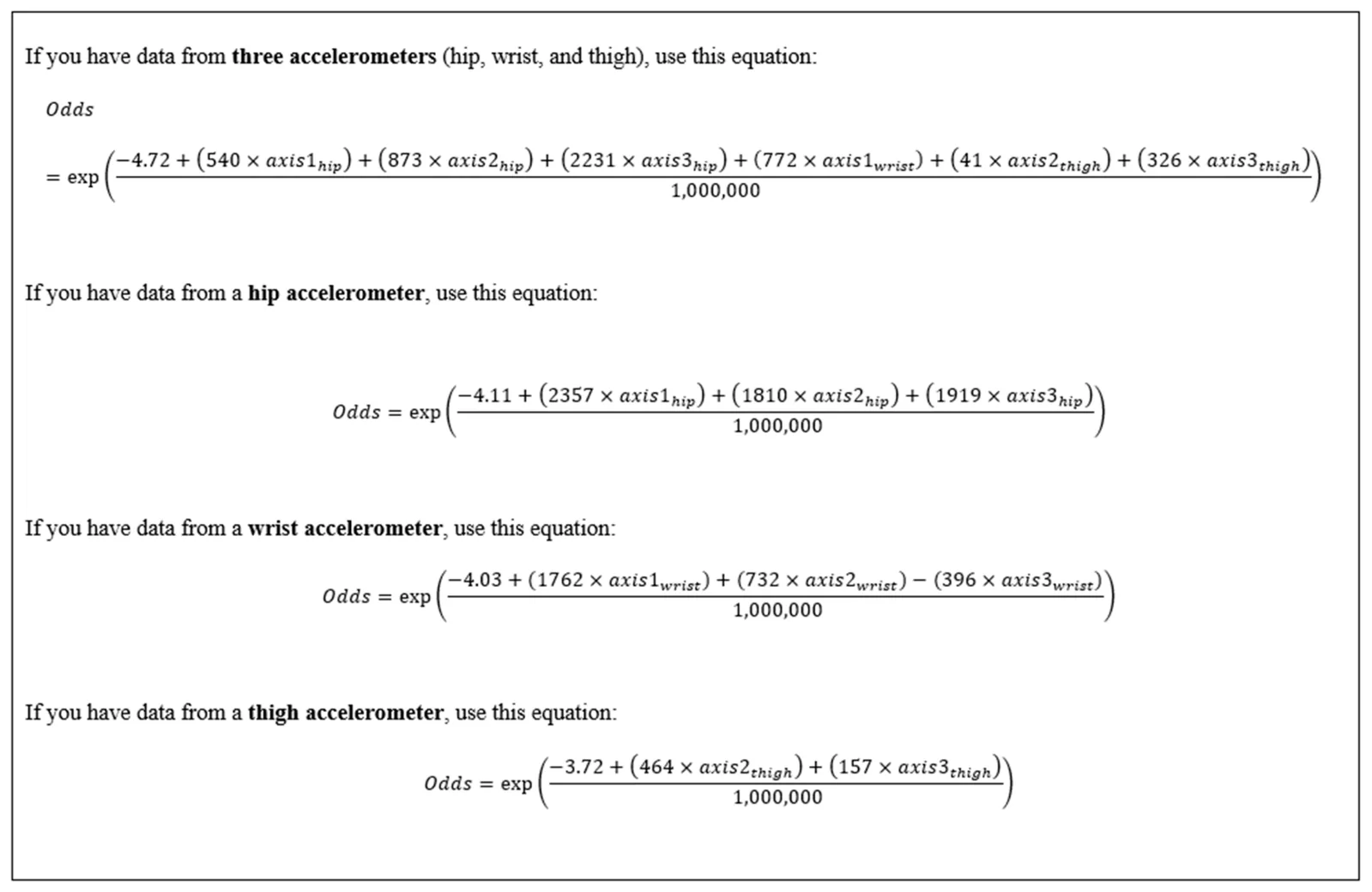
Equations to calculate odds of an epoch being classified as ‘active’.

Those with complete data (from at least one accelerometer) in the development sample (*n* = 21) were entered into the lasso logit process. The CV plot with the selected lambda at minimum CV mean deviance is presented in Supplementary Figure S1, and Supplementary Table S1 shows the CV and goodness-of-fit statistics for the knots from the lasso regression, which indicates that the minimum CV mean deviance for the sample using data from all three accelerometers was 0.2701 for a lambda value of 0.003. This lambda also had the highest out-of-sample deviance ratio of 0.46 and had the lowest BIC for the models with the same number of parameters. The number of non-zero coefficients in the selected model was six. The coefficients from the selected logit model are presented in Supplementary Table S2, and the final equation to generate probabilities that an epoch is part of an activity break is presented in Figure 1.

### 3.2. Classification of Epochs

**Figure 2:**
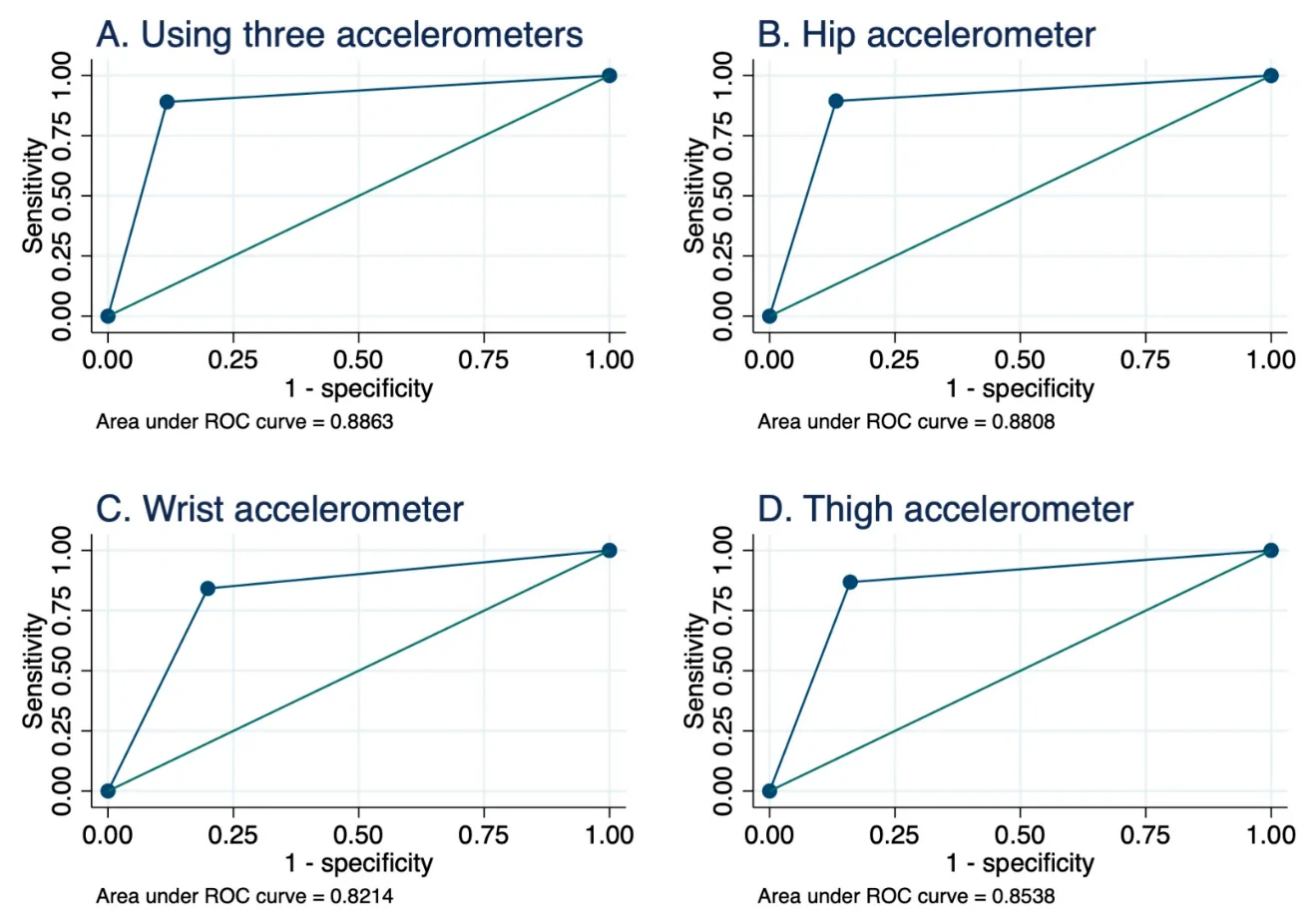
Receiver operating characteristic (ROC) curve showing the sensitivity and specificity of the cut points used to classify epochs as active or sedentary. Cut points are reported in Table 2.

Probabilities were calculated for each epoch according to the prediction model. The Liu method [19] was used to determine that the optimal cut-point probability for classifying the epoch as being in an activity break or not was 0.0515 when data from three accelerometers were used. The ROC curve for this cut point is shown in Figure 2. The sensitivity and specificity of this method for classifying epochs were 0.89 and 0.88, respectively. The AUC was 0.886 (95% CI: 0.879 to 0.894). From all 33,155 epochs, 29,267 (88.3%) were correctly classified.

**Table 2:** Classifying epochs as active or sedentary using probabilities estimated from prediction equation ^a^.

Accelerometer	Total Number of Epochs	Number of Epochs from an Activity Break	Cut-Point ^b^ to Classify Epochs	Number of Epochs Identified as Active	Sensitivity, %	Specificity, %	AUC (95% CI)
All three	33,155	1898	0.0515	5370	89.0	88.2	0.886 (0.879, 0.894)
Hip	35,553	2049	0.0406	6272	89.4	86.8	0.881 (0.874, 0.888)
Wrist	35,553	2049	0.0475	8394	84.2	80.3	0.821 (0.813, 0.830)
Thigh	33,155	1898	0.0408	6673	86.8	83.9	0.854 (0.846, 0.862)

^a^ Coefficients for prediction equations are reported in Supplementary Table S2 and in Figure 1 and Figure 3. ^b^ Epochs with probabilities greater than the cut point are classified as active; otherwise, they are sedentary. Note that further processing is required to identify activity breaks from active epochs; these steps are outlined in Figure 1 and Figure 3.

**Figure 3:**
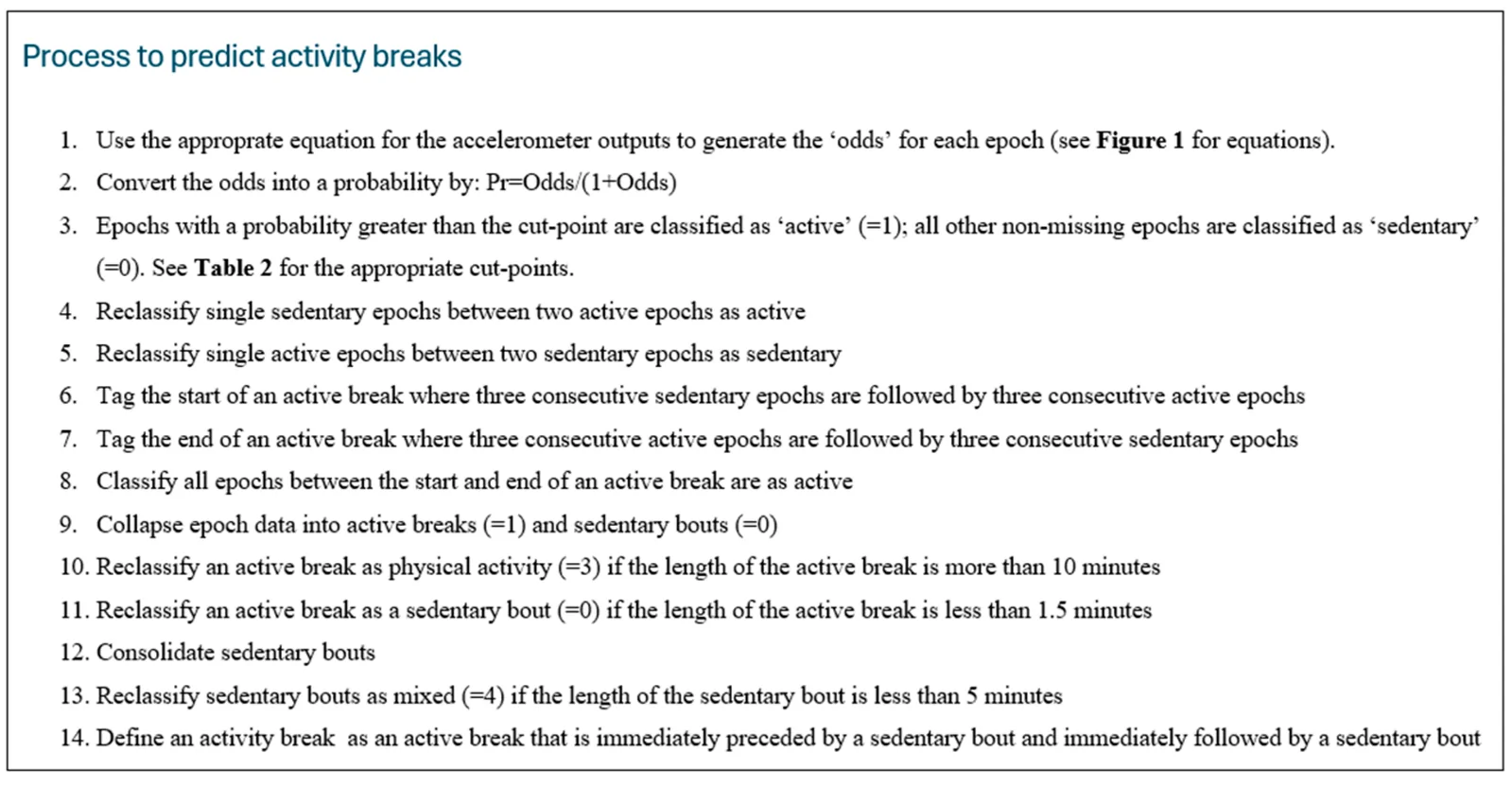
Process to predict activity breaks using accelerometry data. Stata code for this process is provided in Supplementary Materials.

### 3.3. Classification of Activity Breaks

When single ‘active’ epochs (surrounded by ‘sedentary’ epochs) were changed to be sedentary, followed by single sedentary epochs changed to be active epochs, the AUC increased to 0.893. When the order of this process was switched around (single sedentary epochs changed before single active epochs), the AUC was 0.895. Therefore, the latter method was used as it resulted in a higher AUC.

At this stage, there were 471 ‘sedentary bouts’ and 459 ‘active breaks’ altogether in the development sample using data from all three accelerometers. Twenty active breaks from nine participants were then reclassified as ‘physical activity’ because they lasted longer than 10 min (ranging from 10.25 to 41.5 min). There were 180 active breaks that were less than 1.5 min long, and these were reclassified as sedentary. Sedentary spells were reconsolidated after this step, and 83 sedentary bouts less than 5 min were reclassified as ‘mixed’. At this point, there were 211 sedentary bouts and 279 active breaks. The final step defined that, to be an activity break, an active break needed to occur immediately after and immediately before sedentary bouts, which resulted in 144 activity breaks.

The proposed process to predict activity breaks is described in Figure 3, with Stata code provided in the Supplementary Materials. When the predicted number of activity breaks in the development sample was compared to the reported number of activity breaks, the mean difference was less than half an activity break (−0.3, 95% CI: −1.5 to 0.9), with the predicted activity breaks being, on average, 0.5 (95% CI: 0.0 to 1.0) minutes longer.

### 3.4. Classification of Activity Breaks

The process described in Figure 3 was followed using the data from the test sample, which was not used in the development of the process. The mean difference in the number of predicted activity breaks using data from all three accelerometers compared to the number of reported activity breaks was 0.0 (95% CI: −1.7 to 1.7), with the average length of the activity breaks predicted to be 0.9 (95% CI: 0.2 to 1.6) minutes longer than reported.

### 3.5. Different Inputs

The model development and testing process was repeated using the data from each accelerometer (hip, wrist, and thigh) separately. This resulted in three further prediction models if data from only one accelerometer is available. Results from the prediction modelling are reported in Supplementary Figure S1 and Supplementary Tables S1 and S2. The cut points used to classify epochs as active are reported in Table 2. These indicate that the hip accelerometer performed better at predicting active epochs from activity breaks than the other two accelerometers by themselves, with sensitivity and specificity values of 89.4% and 86.8%, respectively. Table 3 shows that the hip accelerometer also did reasonably well at estimating the number of activity breaks when compared to self-report, with a mean difference of 0.0 (−1.5, 1.5) in the development sample, and 0.7 (−1.2, 2.6) in the test sample.

**Figure 4:**
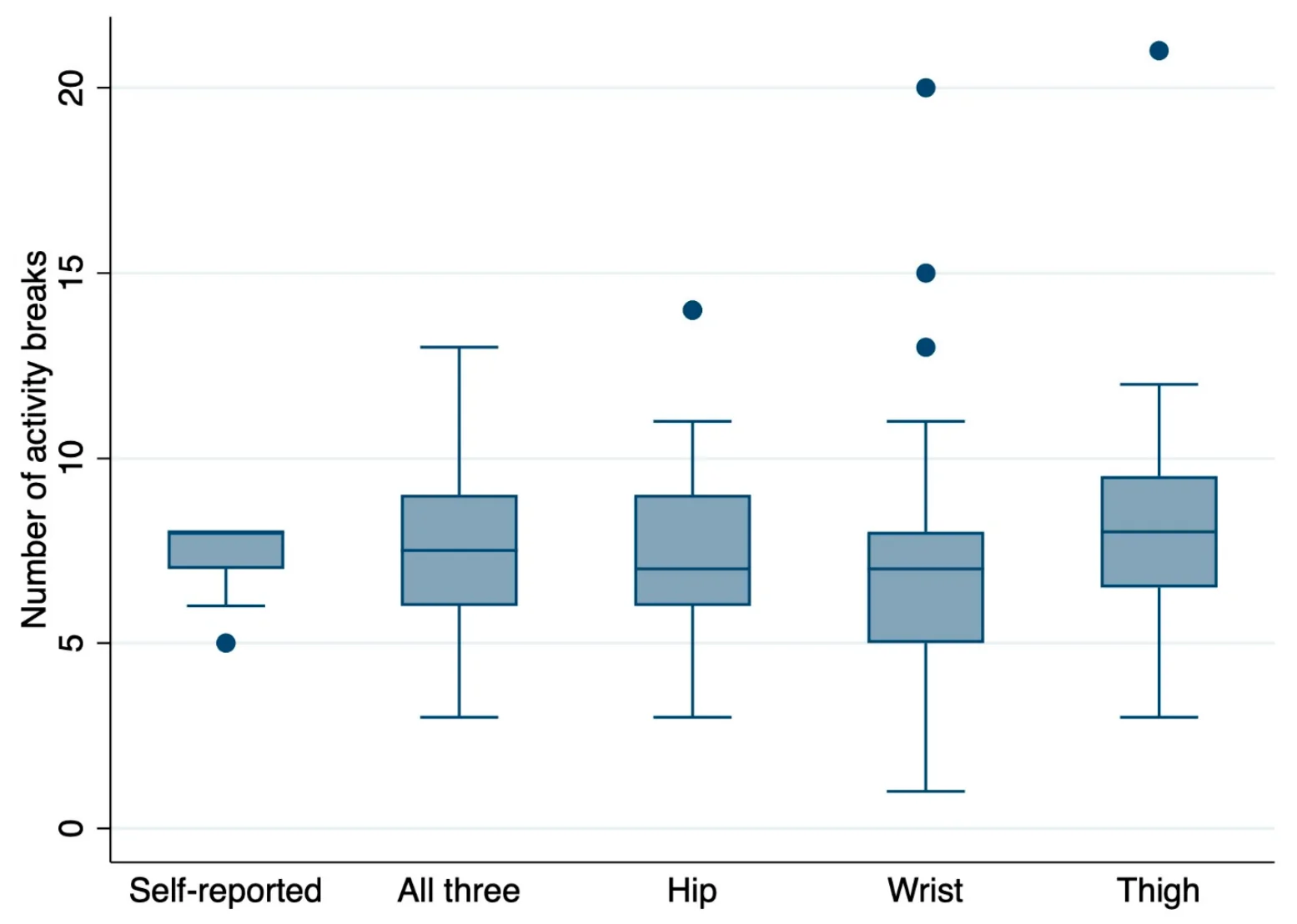
Box plots of number of activity breaks by self-report (*n* = 31), using data from all three accelerometers (*n* = 28), or using data from hip (*n* = 31), wrist (*n* = 31), or thigh (*n* = 28) accelerometers.

Figure 4 illustrates the distributions of predicted activity breaks against the self-reported data and shows that the hip accelerometer was similar to using data from all three accelerometers, with the wrist and thigh accelerometers having a wider distribution and more outliers.

The validity of this algorithm to estimate the mean number and length of activity breaks was strongly supported by external data (Supplementary Table S3), with negligible mean differences between algorithm-determined activity breaks (0.0 (95% CI: −1.1 to 1.0) and self-reported activity breaks (−0.2 (95% CI:−1.0 to 0.7)). However, there was variability in this agreement, as evidenced by the 95% limits of agreement.

## 4. Discussion

We have developed an algorithm that identifies both the number and duration of activity breaks (short (2–3 min), regular (every 20–30 min) bouts of activity) from accelerometer data in healthy adults. The use of this algorithm will allow for accurate identification of intentionally performed activity breaks (such as those that might be promoted in an intervention study) in free-living populations. This is important as research in this area is moving beyond laboratory studies, which have shown that activity breaks in highly controlled environments lead to acute improvements in markers of cardiometabolic health [5]. The focus is now shifting to developing strategies that support this behaviour in free-living settings, where large disparities exist between self-reported and objectively identified breaks in sitting time (used as a proxy for activity breaks) [15]. For example, participants in one study reported taking an average of 10.24 activity breaks per day, whereas accelerometer-based analysis using a sitting-interruption cut point identified only 1.78 breaks per day [15]. This large difference is unlikely to be due solely to social desirability bias, and the results of laboratory studies would indicate that the pattern (rather than just total accumulation) plays an important role in health [21].

To provide the most accurate identification of activity breaks, the algorithm was initially developed using three accelerometers in standard placements on the body. However, we recognise that the participant (and investigator) burden involved in wearing and processing data from three different devices may limit the use of the full version of the algorithm. Thus, prediction algorithms for each accelerometer individually were also developed. The results indicate that while all three prediction models are probably acceptable, the use of the prediction model with the ActiGraph accelerometer placed on the hip produces the greatest sensitivity and specificity, while using the prediction models with the ActiGraph worn on the wrist or the ActivPAL worn on the thigh produces a wider distribution and more outliers. This result may be somewhat unexpected but can be, at least partly, explained by the fact that the ActivPAL tends not to do so well at identifying the intensity of activity [12], while the ActiGraph worn on the wrist does tend to underestimate sedentary time and overestimate time in moderate-to-vigorous physical activity [22]. It is also possible that the types of activity breaks being performed (54% involved walking, running or jogging) may have also contributed to why the hip-worn accelerometer outperformed the other two placements.

As part of the development of the algorithm using all accelerometer data, we performed an external validation using data collected as part of a pilot study of a regular activity breaks intervention, in which participants were asked to perform 2–3 min of activity after every 30 min of uninterrupted sitting in the evening at home. The algorithm performed well in terms of its ability to estimate the mean number of activity breaks (which is likely to be the primary outcome in studies aimed at getting participants to perform activity breaks) in both the test and external samples. However, it performed slightly less well when estimating the length of the activity breaks, overestimating the mean duration by 0.9 min in the test sample, and underestimating by 0.2 min in the external sample. Participants in the external validation sample were not informed that their self-reported activity breaks would later be validated against accelerometer data. The increased variability in this group may therefore be attributable to greater social desirability and recall bias. In contrast, participants in the internal validation sample were aware of the importance of recording activity breaks as accurately as possible, which may have reduced reporting errors. It is also possible that the higher BMI of the external validation sample is contributing to differences, as BMI has been found to influence the accuracy of the prediction equations [23], possibly due to adiposity dampening movement signalling and/or leading to accelerometer placement further away from the centre of mass. While further external validation of the algorithm in a wider range of population demographics may be useful, some level of error is expected with any algorithm. The one developed here appears to perform well in estimating the mean number and duration of activity breaks, which would be the primary outcome in studies aiming to increase this behaviour pattern. However, we advise caution when using the algorithm to estimate the number or duration of activity breaks at an individual level.

### Strengths and Limitations

This algorithm was developed and externally validated in samples of healthy adults who were specifically recruited based on their ability to perform activity breaks of their choosing; it may not be appropriate to use in older adults, adults of differing physical abilities, or children and adolescents—particularly given the use of age-specific cut points and algorithms used to categorise accelerometer output into intensities of activity. However, using the information provided here, these algorithms could easily be adapted for these population groups. We also chose to develop these algorithms using count data rather than raw acceleration. Most accelerometer analysis is still performed using count data [24] and count data appears to be more comparable than raw acceleration data between models of accelerometers [25], possibly making these algorithms more useful and easy to adapt for a variety of other devices. We used self-reported activity break times as the reference method. This approach relied on participants accurately recording the start and end times of their activity breaks, which may have introduced error into the model development. However, most participants were university students or staff and were therefore likely to appreciate the importance of accurate recording. Furthermore, recorded activity breaks did not always occur at the prescribed 30 min intervals, suggesting that participants were recording the actual timing of their movements rather than reporting behaviour that aligned with study instructions. Reduces concerns that responses were influenced by social desirability bias.

The strengths of this study include the use of self-selected activity breaks as the criterion. While many laboratory studies have demonstrated that walking breaks provide clear benefits to cardiometabolic health [5], the use of other types of breaks, particularly resistance exercises, is becoming increasingly popular [26,27] and have been shown to provide similar benefits [27]. Additionally, participants report boredom when asked to perform the same activity breaks on every occasion [28] and therefore it is likely that in a free living setting participants will vary the activities performed. The use of sophisticated statistical modelling techniques to develop a sensitive and specific algorithm that identifies both number and duration of activity breaks is also a strength of this research. While the primary algorithm uses output from multiple accelerometers, we have also provided prediction models for individual accelerometers. The ActiGraph accelerometer worn on the hip, which is still the most commonly used placement in research measuring activity [24], performs the best.

## 5. Conclusions

We have developed algorithms that successfully identify the mean number and duration of activity breaks performed in a free-living setting using accelerometer data. The use of these algorithms will substantially reduce the discrepancies between self-reported and accelerometer-derived estimates of activity breaks. This advancement opens new opportunities for more objective identification of this pattern of activity in large-scale intervention studies.

## Figures and Tables

**Table 1:** Participant characteristics ^a^ (*n* = 31).

	All
Age, y	33 (12)
Gender, *n* (%)	
Male	9 (29)
Female	22 (71)
Ethnicity, *n* (%)	
New Zealand European	25 (81)
Other	6 (19)
Anthropometric measures	
Weight, kg	76 (18)
Height, cm	168 (17)
BMI, kg/m^2^	27 (7)

^a^ Values reported as mean (standard deviation), unless otherwise stated.

**Table 3:** Comparison of observed and predicted activity breaks in the development and test samples.

	Development Sample			Test Sample		
	Median (25th, 75th Percentile) Self-Reported	Median (25th, 75th Percentile) Predicted	Mean Difference (95% CI)	Median (25th, 75th Percentile) Self-Reported	Median (25th, 75th Percentile) Predicted	Mean Difference (95% CI)
Using three accelerometers						
*n*	19	19	19	9	9	9
Number of activity breaks	8 (7, 8)	8 (5, 9)	−0.3 (−1.5, 0.9)	8 (8, 8)	7 (6, 9)	0.0 (−1.7, 1.7)
Mean length of activity breaks, minutes	3.0 (2.8, 3.4)	3.4 (2.9, 3.9)	0.5 (0.0, 1.0)	2.3 (2.3, 2.6)	3.2 (2.8, 3.4)	0.9 (0.2, 1.6)
Hip accelerometer						
*n*	21	21	21	10	10	10
Number of activity breaks	8 (7, 8)	7 (6, 9)	0.0 (−1.5, 1.5)	8 (8, 8)	8 (7, 10)	0.7 (−1.2, 2.6)
Mean length of activity breaks, minutes	2.9 (2.8, 3.1)	3.4 (3.1, 4.2)	0.6 (0.3, 1.0)	2.4 (2.3, 3.0)	3.2 (2.8, 3.7)	0.7 (0.2, 1.3)
Wrist accelerometer						
*n*	21	21	21	10	10	10
Number of activity breaks	8 (7, 8)	7 (6, 8)	0.2 (−1.7, 2.1)	8 (8, 8)	7 (5, 8)	−0.3 (−2.6, 2.0)
Mean length of activity breaks, minutes	2.9 (2.8, 3.1)	3.8 (3.2, 4.1)	0.6 (0.2, 1.0)	2.4 (2.3, 3.0)	3.5 (2.7, 4.8)	1.2 (0.4, 2.1)
Thigh accelerometer						
*n*	19	19	19	9	9	9
Number of activity breaks	8 (7, 8)	8 (7, 9)	0.7 (−1.2, 2.6)	8 (8, 8)	8 (5, 10)	−0.2 (−2.1, 1.7)
Mean length of activity breaks, minutes	3.0 (2.8, 3.4)	3.9 (3.1, 4.4)	0.8 (0.3, 1.3)	2.3 (2.3, 2.6)	3.1 (2.7, 3.2)	0.8 (0.1, 1.5)

## Data Availability

Data may be made available upon reasonable request to the corresponding author.

## Supplementary Materials

The following supporting information can be downloaded at https://www.mdpi.com/article/10.3390/s26196194/s1, Figure S1: Cross-validation (CV) plots of the CV mean deviance on the y-axis and lambda values on the x-axis. The lambda with the lowest CV mean deviance is indicated by the vertical line and is reported in Supplementary Table S1; Table S1: Cross-validation and goodness-of-fit statistics for the knots from lasso regressions using development sample data from three accelerometers together (*n* = 19) and from each accelerometer at the hip (*n* = 21), wrist (*n* = 21), and thigh (*n* = 19); Table S2: Non-zero coefficients for the prediction models selected by lasso logit regression; Table S3: External validation assessment of algorithm compared to self-reported activity breaks in the evening (*n* = 20); Data S1: Stata code to identify activity breaks using data from three accelerometers (A), or hip (B), wrist (C), or thigh accelerometers (D).

## Abbreviations

The following abbreviations are used in this manuscript:

AUC	Area under the curve
BMI	Body mass index
CV	Cross validation
ROC	Receiver operating characteristic
SD	Standard deviation
