# Supplementary material for: Identification of Activity Breaks Using Accelerometry
Source: Sensors (Basel). 2026 Sep 30;26(19):6194. doi: 10.3390/s26196194 (PMC13667502; doi:10.3390/s26196194)
Supplement: Supplementary file 1 [file sensors-26-06194-s001.zip › sensors-4493857-supplementary.pdf]

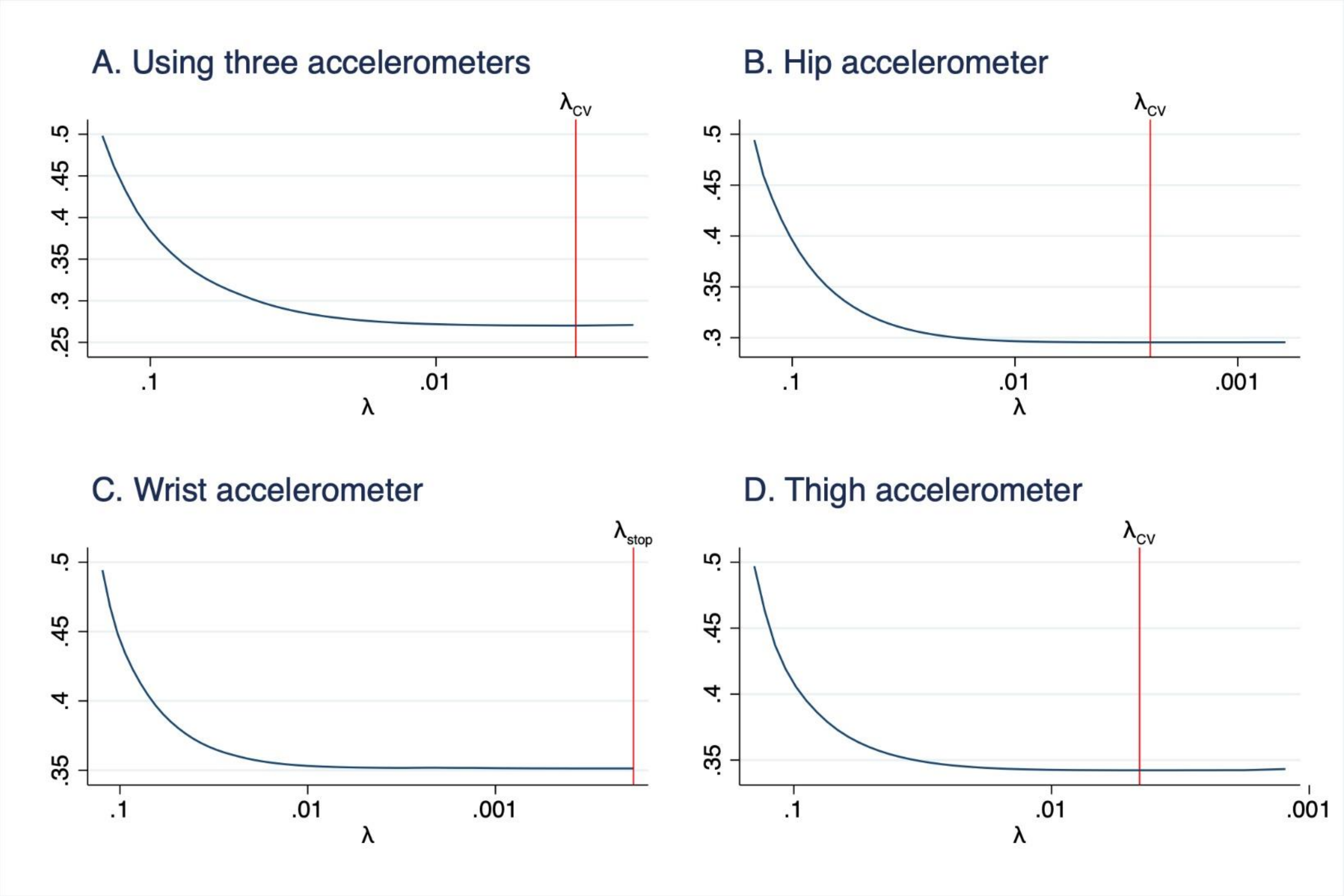

Supplementary Figure S1. Cross-validation (CV) plots of the CV mean deviance on the y-axis and lambda values on the x-axis. The lambda with the lowest CV mean deviance is indicated by the vertical line and is reported in Supplementary Table S1.

Supplementary Table S1. Cross-validation and goodness-of-fit statistics for the knots from lasso regressions using development sample data from three accelerometers together (n=19) and from each accelerometer at the hip (n=21), wrist (n=21), and thigh (n=19)

Lambda knots	Cross-validation mean deviance	Out-of-sample deviance ratio	BIC	Number of non-zero coefficients
**Using three accelerometers**				
0.134	0.4612	0.0768	17.6	2
0.122	0.4324	0.1346	19.9	3
0.111	0.4070	0.1853	22.3	4
0.101	0.3871	0.2253	24.9	5
0.053	0.3127	0.3742	26.3	6
0.003[a]	0.2701	0.4593	25.5	6
0.002	0.2709	0.4578	25.5	6
**Hip accelerometer**				
0.135	0.4597	0.0744	15.7	1
0.123	0.4365	0.1210	18.2	2
0.093	0.3842	0.2264	20.1	3
0.002[a]	0.2955	0.4051	18.2	3
0.001	0.2956	0.4049	18.2	3
**Wrist accelerometer**				
0.113	0.4681	0.0575	15.8	1
0.049	0.3803	0.2341	17.1	2
0.025[a]	0.3518	0.2916	19.5	3
0.0002	0.3513	0.2926	19.5	3
**Thigh accelerometer**				
0.1297	0.4632	0.0729	14.6	1
0.1077	0.4191	0.1612	16.7	2
0.005[a]	0.3423	0.3149	15.2	2
0.001	0.3432	0.3130	18.1	3

[a]The lambda for the final model was selected at the minimum cross-validation mean deviance

Supplementary Table S2. Non-zero coefficients for the prediction models selected by lasso logit regression

	Coefficients from model with data from three accelerometers	Coefficients from model with data from hip accelerometer	Coefficients from model with data from wrist accelerometer	Coefficients from model with data from thigh accelerometer
**Hip**				
Axis 1	0.000540	0.002357	NA	NA
Axis 2	0.000873	0.001810	NA	NA
Axis 3	0.002231	0.001919	NA	NA
**Wrist**				
Axis 1	0.000772	NA	0.001762	NA
Axis 2	NA	NA	0.000732	NA
Axis 3	NA	NA	-0.000396	NA
**Thigh**				
Axis 1	NA	NA	NA	NA
Axis 2	0.000041	NA	NA	0.000464
Axis 3	0.000326	NA	NA	0.000157
**Constant**	-4.72	-4.11	-4.03	-3.72

NA, not applicable. These variables are not included in the model.

Supplementary Table S3. External validation assessment of algorithm compared to self-reported activity breaks in the evening (n=20)

	Self-reported activity break, Mean (SD)	Algorithm-defined activity break, Mean (SD)	Mean difference (95% CI) algorithm compared to self-reported	p-value	95% limits of agreement
First three days of RAB intervention					
Mean number of breaks	3.2 (1.7)	3.2 (1.8)	0.0 (-1.1, 1.1)	0.938	-4.7 to 4.8
Mean length of breaks, min	3.1 (0.8)	2.9 (1.2)	-0.2 (-0.9, 0.5)	0.589	-3.3 to 2.9
Last three days of RAB intervention					
Mean number of breaks	2.8 (1.5)	2.8 (2.2)	0.0 (-1.0, 1.1)	0.929	-4.2 to 4.3
Mean length of breaks, min	2.5 (1.1)	2.3 (1.6)	-0.2 (-1.0, 0.7)	0.939	-3.7 to 3.3

Supplementary Data S1. Stata code to identify activity breaks using data from three accelerometers (A), or hip (B), wrist (C), or thigh accelerometers (D).

A. Stata code to determine activity breaks using three accelerometers:

```
/*
Three hip accelerometer variables should be named: 'axis1_hip', 'axis2_hip', 'axis3_hip'
Three wrist accelerometer variables should be named: 'axis1_wrist', 'axis2_wrist'
Three thigh accelerometer variables should be named: 'axis2_thigh', 'axis3_thigh'
'axis 1' is the y-axis; 'axis 2' is the x-axis; 'axis 3' is the z-axis.
Epochs should be numbered and the variable named 'epoch'
Participant id variable named 'id'
If not installed run: ssc install tsspell
Paste this code into a do-file.
*/

//Using the data from the accelerometers, calculate the odds
gen odds=exp(-4.72 +((540*axis1_hip)+(873*axis2_hip)+(2231*axis3_hip) ///
+(772*axis1_wrist) +(41*axis2_thigh)+(326*axis3_thigh))/1000000)
//Calculate the probability that the epoch is active
gen pr=odds/(1+odds)
//Classify into active break or not
gen ab=0
replace ab=1 if pr>0.0515
//Sort the epochs
sort id epoch
//Make single 0s into 1s; then single 1s into 0s
replace ab=1 if ab[_n-1]==1 & ab[_n+1]==1
replace ab=0 if ab[_n-1]==0 & ab[_n+1]==0
//Create start and finish variables if there is a switch between 3 consecutive 1s and 0s
gen start_ab=0
replace start_ab=1 if ab==0 & ab[_n+1]==1 & ab[_n+2]==1 & ab[_n+3]==1 ///
& ab[_n-1]==0 & ab[_n-2]==0
gen finish_ab=0
replace finish_ab=1 if ab==0 & ab[_n-1]==1 & ab[_n-2]==1 & ab[_n-3]==1 ///
& ab[_n+1]==0 & ab[_n+2]==0
//Fill in the active break with 1s
gen ab_total=0
replace ab_total=1 if start_ab[_n-1]==1
replace ab_total=1 if finish_ab[_n+1]==1
replace ab_total=1 if ab_total[_n-1]==1 & finish_ab!=1
//Collapse active breaks and sedentary bouts using the tsspell package
tsset id epoch
tsspell ab_total
sort id _spell epoch
by id _spell: egen length = max(_seq)
//Reduce dataset
keep id _spell ab_total length
duplicates drop
//Define a length of activity of greater than 10 minutes (40 epochs) as 'physical
activity' (=3)
replace ab_total=3 if ab_total==1 & length>40
//Define a length of activity less than 1.5 minutes (6 epochs) as sedentary:
replace ab_total=0 if ab_total==1 & length<6
rename _spell spell
//Collapse together the new sedentary classifications
tsset id spell
tsspell ab_total
sort id _spell
by id _spell: egen newlength = total(length)
keep id ab_total _spell  newlength
duplicates drop
//Define a sedentary time less than 5 mins as mixed (=4)
replace ab_total=4 if ab_total!=1 & newlength<20
//Define an activity break as an active break between 6 and 40 epochs, between two
sedentary bouts >5 mins
sort id _spell
gen ab=1 if ab_total==1 & ab_total[_n-1]==0 & ab_total[_n+1]==0
//Create a dataset of number and length of activity breaks
egen ab_no=count(ab), by(id)
egen ab_length=mean(newlength) if ab==1, by(id)
keep id ab_no ab_length
duplicates drop
//Convert activity break length from epochs into minutes
gen ab_length_min=ab_length/4
```

B. Stata code to determine activity breaks using data from a hip accelerometer:

```
/*
Three hip accelerometer variables should be named: 'axis1_hip', 'axis2_hip', 'axis3_hip'
Epochs should be numbered and the variable named 'epoch'
Participant id variable named 'id'
If not installed run: ssc install tsspell
Paste this code into a do-file.
*/

//Using the data from the accelerometers, calculate the odds
gen odds=exp(-4.11 + ((2357*axis1_hip)+(1810*axis2_hip)+(1919*axis3_hip))/1000000)
//Calculate the probability that the epoch is active
gen pr=odds/(1+odds)
//Classify into active break or not
gen ab=0
replace ab=1 if pr>0.0406
//Sort the epochs
sort id epoch
//Make single 0s into 1s; then single 1s into 0s
replace ab=1 if ab[_n-1]==1 & ab[_n+1]==1
replace ab=0 if ab[_n-1]==0 & ab[_n+1]==0
//Create start and finish variables if there is a switch between 3 consecutive 1s and 0s
gen start_ab=0
replace start_ab=1 if ab==0 & ab[_n+1]==1 & ab[_n+2]==1 & ab[_n+3]==1 ///
& ab[_n-1]==0 & ab[_n-2]==0
gen finish_ab=0
replace finish_ab=1 if ab==0 & ab[_n-1]==1 & ab[_n-2]==1 & ab[_n-3]==1 ///
& ab[_n+1]==0 & ab[_n+2]==0
//Fill in the active break with 1s
gen ab_total=0
replace ab_total=1 if start_ab[_n-1]==1
replace ab_total=1 if finish_ab[_n+1]==1
replace ab_total=1 if ab_total[_n-1]==1 & finish_ab!=1
//Collapse active breaks and sedentary bouts using the tsspell package
tsset id epoch
tsspell ab_total
sort id _spell epoch
by id _spell: egen length = max(_seq)
//Reduce dataset
keep id _spell ab_total length
duplicates drop
//Define a length of activity of greater than 10 minutes (40 epochs) as 'physical
activity' (=3)
replace ab_total=3 if ab_total==1 & length>40
//Define a length of activity less than 1.5 minutes (6 epochs) as sedentary:
replace ab_total=0 if ab_total==1 & length<6
rename _spell spell
//Collapse together the new sedentary classifications
tsset id spell
tsspell ab_total
sort id _spell
by id _spell: egen newlength = total(length)
keep id ab_total _spell  newlength
duplicates drop
//Define a sedentary time less than 5 mins as mixed (=4)
replace ab_total=4 if ab_total!=1 & newlength<20
//Define an activity break as an active break between 6 and 40 epochs, between two
sedentary bouts >5 mins
sort id _spell
gen ab=1 if ab_total==1 & ab_total[_n-1]==0 & ab_total[_n+1]==0
//Create a dataset of number and length of activity breaks
egen ab_no=count(ab), by(id)
egen ab_length=mean(newlength) if ab==1, by(id)
keep id ab_no ab_length
duplicates drop
//Convert activity break length from epochs into minutes
gen ab_length_min=ab_length/4
```

C. Stata code to determine activity breaks using data from a wrist accelerometer:

```
/*
Three hip accelerometer variables should be named: 'axis1_wrist', 'axis2_wrist',
'axis3_wrist'
Epochs should be numbered and the variable named 'epoch'
Participant id variable named 'id'
If not installed run: ssc install tsspell
Paste this code into a do-file.
*/

//Using the data from the accelerometers, calculate the odds
gen odds=exp(-4.03 + ((1762*axis1_wrist)+(732*axis2_wrist)-(396*axis3_wrist))/1000000))
//Calculate the probability that the epoch is active
gen pr=odds/(1+odds)
//Classify into active break or not
gen ab=0
replace ab=1 if pr>0.0475
//Sort the epochs
sort id epoch
//Make single 0s into 1s; then single 1s into 0s
replace ab=1 if ab[_n-1]==1 & ab[_n+1]==1
replace ab=0 if ab[_n-1]==0 & ab[_n+1]==0
//Create start and finish variables if there is a switch between 3 consecutive 1s and 0s
gen start_ab=0
replace start_ab=1 if ab==0 & ab[_n+1]==1 & ab[_n+2]==1 & ab[_n+3]==1 ///
& ab[_n-1]==0 & ab[_n-2]==0
gen finish_ab=0
replace finish_ab=1 if ab==0 & ab[_n-1]==1 & ab[_n-2]==1 & ab[_n-3]==1 ///
& ab[_n+1]==0 & ab[_n+2]==0
//Fill in the active break with 1s
gen ab_total=0
replace ab_total=1 if start_ab[_n-1]==1
replace ab_total=1 if finish_ab[_n+1]==1
replace ab_total=1 if ab_total[_n-1]==1 & finish_ab!=1
//Collapse active breaks and sedentary bouts using the tsspell package
tsset id epoch
tsspell ab_total
sort id _spell epoch
by id _spell: egen length = max(_seq)
//Reduce dataset
keep id _spell ab_total length
duplicates drop
//Define a length of activity of greater than 10 minutes (40 epochs) as 'physical
activity' (=3)
replace ab_total=3 if ab_total==1 & length>40
//Define a length of activity less than 1.5 minutes (6 epochs) as sedentary:
replace ab_total=0 if ab_total==1 & length<6
rename _spell spell
//Collapse together the new sedentary classifications
tsset id spell
tsspell ab_total
sort id _spell
by id _spell: egen newlength = total(length)
keep id ab_total _spell  newlength
duplicates drop
//Define a sedentary time less than 5 mins as mixed (=4)
replace ab_total=4 if ab_total!=1 & newlength<20
//Define an activity break as an active break between 6 and 40 epochs, between two
sedentary bouts >5 mins
sort id _spell
gen ab=1 if ab_total==1 & ab_total[_n-1]==0 & ab_total[_n+1]==0
//Create a dataset of number and length of activity breaks
egen ab_no=count(ab), by(id)
egen ab_length=mean(newlength) if ab==1, by(id)
keep id ab_no ab_length
duplicates drop
//Convert activity break length from epochs into minutes
gen ab_length_min=ab_length/4
```

D. Stata code to determine activity breaks using data from a thigh accelerometer:

```
/*
Three hip accelerometer variables should be named: ‘axis1_thigh’, ‘axis2_thigh’,
‘axis3_thigh’
Epochs should be numbered and the variable named ‘epoch’
Participant id variable named ‘id’
If not installed run: ssc install tsspell
Paste this code into a do-file.
*/

//Using the data from the accelerometers, calculate the odds
gen odds=exp(-3.72 + ((464*axis2_thigh)+(157*axis3_thigh))/1000000))
//Calculate the probability that the epoch is active
gen pr=odds/(1+odds)
//Classify into active break or not
gen ab=0
replace ab=1 if pr>0.0408
//Sort the epochs
sort id epoch
//Make single 0s into 1s; then single 1s into 0s
replace ab=1 if ab[_n-1]==1 & ab[_n+1]==1
replace ab=0 if ab[_n-1]==0 & ab[_n+1]==0
//Create start and finish variables if there is a switch between 3 consecutive 1s and 0s
gen start_ab=0
replace start_ab=1 if ab==0 & ab[_n+1]==1 & ab[_n+2]==1 & ab[_n+3]==1 ///
& ab[_n-1]==0 & ab[_n-2]==0
gen finish_ab=0
replace finish_ab=1 if ab==0 & ab[_n-1]==1 & ab[_n-2]==1 & ab[_n-3]==1 ///
& ab[_n+1]==0 & ab[_n+2]==0
//Fill in the active break with 1s
gen ab_total=0
replace ab_total=1 if start_ab[_n-1]==1
replace ab_total=1 if finish_ab[_n+1]==1
replace ab_total=1 if ab_total[_n-1]==1 & finish_ab!=1
//Collapse active breaks and sedentary bouts using the tsspell package
tsset id epoch
tsspell ab_total
sort id _spell epoch
by id _spell: egen length = max(_seq)
//Reduce dataset
keep id _spell ab_total length
duplicates drop
//Define a length of activity of greater than 10 minutes (40 epochs) as ‘physical
activity’ (=3)
replace ab_total=3 if ab_total==1 & length>40
//Define a length of activity less than 1.5 minutes (6 epochs) as sedentary:
replace ab_total=0 if ab_total==1 & length<6
rename _spell spell
//Collapse together the new sedentary classifications
tsset id spell
tsspell ab_total
sort id _spell
by id _spell: egen newlength = total(length)
keep id ab_total _spell  newlength
duplicates drop
//Define a sedentary time less than 5 mins as mixed (=4)
replace ab_total=4 if ab_total!=1 & newlength<20
//Define an activity break as an active break between 6 and 40 epochs, between two
sedentary bouts >5 mins
sort id _spell
gen ab=1 if ab_total==1 & ab_total[_n-1]==0 & ab_total[_n+1]==0
//Create a dataset of number and length of activity breaks
egen ab_no=count(ab), by(id)
egen ab_length=mean(newlength) if ab==1, by(id)
keep id ab_no ab_length
duplicates drop
//Convert activity break length from epochs into minutes
gen ab_length_min=ab_length/4
```
